# *Asellus aquaticus* as a Potential Carrier of *Escherichia coli* and Other Coliform Bacteria into Drinking Water Distribution Systems 

**DOI:** 10.3390/ijerph10030845

**Published:** 2013-03-01

**Authors:** Sarah C. B. Christensen, Erik Arvin, Erling Nissen, Hans-Jørgen Albrechtsen

**Affiliations:** 1 DTU Environment, Technical University of Denmark, Bygningstorvet B115, 2800 Kgs. Lyngby, Denmark; E-Mails: erar@env.dtu.dk (E.A.); hana@env.dtu.dk (H.-J.A.); 2 VCS Denmark, Vandværksvej 7, 5000 Odense C., Denmark; E-Mail: en@vandcenter.dk

**Keywords:** asellids, fresh water, indicator organisms, invertebrate, microbial, water louse

## Abstract

Individuals of the water louse, *Asellus aquaticus,* enter drinking water distribution systems in temperate parts of the world, where they establish breeding populations. We analysed populations of surface water *A. aquaticus* from two ponds for associated faecal indicator bacteria and assessed the risk of *A. aquaticus* transporting bacteria into distribution systems. Concentrations of up to two *E. coli* and five total coliforms·mL^−1^ were measured in the water and 200 *E. coli* and >240 total coliforms·mL^−1^ in the sediments of the investigated ponds. Concentrations of *A. aquaticus* associated bacteria never exceeded three *E. coli* and six total coliforms·*A. aquaticus*^−1^. During exposure to high concentrations of coliforms, concentrations reached 350 coliforms·*A. aquaticus*^−1^. *A. aquaticus* associated *E. coli* were only detected as long as *E. coli* were present in the water and sediment. The calculated probability of exceeding drinking water guideline values in non-disinfected systems by intrusion of *A. aquaticus* was low. Only in scenarios with narrow pipes and low flows, did total coliforms exceed guideline values, implying that the probability of detection by routine monitoring is also low. The study expands the knowledge base for evaluating incidents with presence of coliform indicators in drinking water by showing that intruding *A. aquaticus* were not important carriers of *E. coli* or other coliform bacteria even when emerging from faecally contaminated waters.

## 1. Introduction

The water louse *Asellus aquaticus* (Isopoda) is frequently found in drinking water distribution systems in temperate parts of the world (e.g., [1,2,3,4]). When *A. aquaticus* or other invertebrate animals enter a drinking water distribution system, they may be contaminated with bacteria, which can affect human health [5]. However, only few studies on this topic have been published and they mainly concern nematodes (e.g., [6,7]). The invertebrates may enter the systems via surface water, ground water or through leaks in clean water tanks and pipes created by bursts or maintenance work [2,8]. During repairs or maintenance work it is good practice to drain the hole around the pipe and exchange the pipe immediately. However, in practice incidences do occur for example during heavy rain events when surface water contaminates the pipe systems. Clean water tanks are often underground, which hinders observation of cracks in walls or ceilings from the outside and surface water animals may enter tanks through cracks, e.g., during heavy precipitations. 

*A. aquaticus* from drinking water distribution systems have eyes and most specimens are coloured brown [1,9] in contrast to subsurface asellids (e.g., *Proasellus cavaticus*) which are transparent and have reduced or no eyes [9,10]. This indicates that even in water supplies based on groundwater, *A. aquaticus* most likely enter from surface water through deficiencies in the system. The public, press and water utilities in Denmark have shown an increasing interest in the occurrence of the animals in drinking water systems and raised concerns regarding drinking water safety.

Naturally occurring populations of *A. aquaticus* can survive at oxygen concentrations as low as 0.3 mg·L^−1^ and are known to thrive in highly eutrophic environments [11], where faecal bacteria are often present. However, *A. aquaticu*s adapt to oligotrophic environments such as drinking water distribution systems, where they are able to form breeding populations and may survive even longer than in nature [1]. *A. aquaticus* is one of the largest invertebrates in drinking water systems. They are of particular interest as potential carriers of bacteria into drinking water systems when coming from contaminated environments, since the amount of bacteria associated with other investigated invertebrates (*Hyalella azteca*) depend on the size of the invertebrate [12]. Furthermore, *A. aquaticus* has been found to harbour bacteria from at least four different genera as symbionts in the digestive glands [13]. 

*Escherichia coli* and total coliform bacteria are widely used as indicators for faecal contaminations and as indicators of water quality in general [14,15] but some *E. coli* strains are also highly virulent (e.g., [16]). Other coliform bacteria can be present in both faeces and the environment (nutrient-rich waters, soil, decaying plant material) and some strains can even grow in drinking water distribution systems [17].

In water supply systems with distribution of drinking water without disinfection or disinfection residual such as in e.g., Denmark [1], the Netherlands [18] and Switzerland [19] there is no hygienic barrier against bacterial contaminations before the water reaches the end users. Therefore, bacteria transported into distribution systems by invertebrates may reach the consumers. In distribution systems with treatments such as chlorination or UV light, invertebrates may protect pathogens against the disinfection agents [12,20].

The aims of this study were to investigate whether *A. aquaticus* from surface waters are carriers of the faecal indicator organism *E. coli* or other coliform bacteria and if so, to estimate the probability of exceeding drinking water guideline values when *A. aquaticus* intrude drinking water distribution systems.

## 2. Materials and Methods

### 2.1. Sampling Locations

Two sampling locations near Kgs. Lyngby, Denmark were investigated. Pond 1 (55°47′17″ N, 12°31′30″ E) is an artificial eutrophic pond of 12 m^2^ approximately 0.3 meter deep with concrete sides and a sediment covered bottom. The vegetation was dominated by reeds, *Nuphar* sp. (waterlily) and *Lemna minor* (common duckweed) and the dominating non-aquatic fauna were waterfowl, mainly ducks. Pond 2 (55°46′57″ N, 12°33′47″ E) is situated in a deer park. It covers an area of 3,000 m^2^ and has a maximum depth of three to four meters, a muddy bottom and vegetation dominated by various reeds, *Elodea canadensis* (pondweed), *Lemna trisulca* (star duckweed) and *Riccia fluitans* (crystalwort). The dominating non-aquatic fauna were deer and waterfowl such as ducks and swans. Pond 2 is categorized as a Natura 2000 habitat 3150 “naturally eutrophic with floating vegetation”. With different fauna groups present in the two ponds, samples of coliform bacteria originated from birds as well as from mammals.

### 2.2. Sampling

Water, sediment and 60–100 *A. aquaticus* (depending on availability) were collected on each sampling occasion from pond 1 in January and October 2010 and from pond 2 in September and October 2010. Water was collected from the surfaces of the ponds 0.5 m from shore while sediment was sampled at 30 cm depth 0.5 m from shore. Water and surface sediment samples for analysis were taken by sterile pipettes and kept in two times five sterile 100 mL plastic containers while *A. aquaticus* were collected by a one mm meshed net and kept in three 500 mL glass containers containing water from the ponds. The average length was 10 mm but in the January samples, large hibernating specimens up to 25 mm were also sampled. All samples were transported to the lab within 15 min and analysed within 2 h. Two litres of pond water for the experiments with *A. aquaticus* were sampled on each sampling occasion in pre-combusted (550 °C for 18 h) blue cap bottles.

### 2.3. Analyses

Sediment, water and 10–20 *A. aquaticus* (depending on availability) from each sampling occasion were analysed for the presence of metabolically active *E. coli* and total metabolically active coliform bacteria by Colilert^®^ (IDEXX Europe BV, Hoofddorp, The Netherlands) following the manufacturer’s instructions. Water and sediment samples were analysed in duplicates and triplicates. Ten mL wet sediment was extracted from the samples with sterile pipettes and dispersed in 90 mL autoclaved tap water before being poured into the Quanti-Tray^®^/2000 trays. One hundred mL of water was used for each analysis of the pond water. Quanti-Tray^®^/2000 trays were incubated at 37 ± 1 °C and after 20 ± 2 h the numbers of yellow wells were used to determine Most Probable Number (MPN) for total coliforms according to IDEXX Quanti-Tray^®^/2000 MPN provided by the manufacturer (IDEXX). The number of fluorescent wells under UV light was used to determine MPN for *E. coli*. 

Prior to analysis, the animals were placed on absorbent tissue to remove attached water, grinded with a sterile pestle in a sterile tray and suspended in one mL autoclaved tap water. Two to four *A. aquaticus* were pooled for each Colilert^®^ analysis. No distinction was made between adhering and internal bacteria, nor has this been studied in the available literature. Since detection by Colilert^®^ relies on an enzymatic reaction, pre-experiments were carried out as described by Christensen *et al.* [21], establishing that grinded *A. aquaticus* did not influence the reaction. The pre-experiments also established that the absorbent tissue prevented bacteria from the water phase from being quantified together with the *A. aquaticus* associated bacteria. Because this study assessed the bacteria carried by *A. aquaticus in vivo*, the surfaces of *A. aquaticus* were not disinfected prior to analysis.

Total culturable heterotrophic bacteria, associated with *A. aquaticus* were measured as heterotrophic plate counts (HPC) on R2A agar (17209 Fluka Analytical, Sigma-Aldrich, Buchs, Switzerland) by plate spreading [22] with incubation at 20 ± 1 °C for 14 days.

### 2.4. Increased Concentrations of Coliform Bacteria

To study the association between *A. aquaticus* and coliform bacteria under increased concentrations of coliform bacteria, *A. aquaticus* from pond 2, September samples (which contained the highest concentrations of *E. coli*), were distributed in three sterile glass beakers (30 *A. aquaticus* in each). The beakers contained 450 mL pond water supplemented with 100 mL tap water (7 ± 1 °C) when 100 mL subsamples were taken, to maintain the volume, and were kept at 7 ± 1 °C for nine weeks. The temperature corresponded to temperatures in Danish distribution systems as well as the approximated average temperature of the ponds during sampling. Increased coliform growth rates would be expected under higher temperatures [23] however, the decay of *E. coli* under low temperatures is low [24]. Maple leaves which had been stored frozen and 50 mL drinking water sediment (wet volume), flushed from a fire hydrant, were added to the systems for *A. aquaticus* to feed on. Subsamples of the applied leaves and drinking water sediment were analysed by Colilert^®^ and did not indicate presence of *E. coli* or other coliform bacteria. 

### 2.5. Calculations

The concentration, C, of the bacteria released from *A. aquaticus* into the drinking water in a pipe was calculated by:

[C = M/Q]
(1)

where M is the source strength [# bacteria released·s^−1^] and Q is the flow rate (m^3^·s^−1^) in the pipe calculated by:

[Q = V × A]
(2)

where V = flow velocity (m·s^−1^) in pipe and A = flow area (m^2^) of the pipe. 

The calculation gives a conservative assessment of the bacteria concentrations immediately after release to the water since it omits dispersion, sedimentation and inactivation which will reduce concentration over time.

## 3. Results and Discussion

### 3.1. A. aquaticus and Associated Coliform Bacteria from Surface Waters

*A. aquaticus* as well as *E. coli* and other coliform bacteria were present in the investigated ponds (Table 1). Only a maximum of three *A. aquaticus* associated *E. coli* and six *A. aquaticus* associated total coliforms were measured despite the relatively high concentrations of *E. coli* and total coliforms in water (two *E. coli*·mL^−1^ and five total coliforms·mL^−1^) and sediment (200 *E. coli*·mL^−1^ and >240 total coliforms·mL^−1^). No *E. coli* or other coliform bacteria were detected on large (25 mm long) hibernating *A. aquaticus* collected in January (Table 1). 

**Table 1:** Concentrations of *E. coli* and total coliform bacteria in water, sediment and associated with *A. aquaticus* from two surface water ponds.

	Pond 1				Pond 2			
	January 2010 0 °C (ice cover)		October 2010 11 °C		September 2010 5 °C		October 2010 6 °C	
	E	T	E	T	E	T	E	T
Water phase (MPN·mL^−1^)	<0.01	0.03	0.01 ± 0.01	5 ± 1.9	2 ± 1.4	5 ± 1.9	0.1 ± 0.03	1 ± 0.05
Sediment phase (MPN·mL^−1^)	<1	<1	14 ± 9.7	>240	200 ± 6	>240	>86	>170
Associated (MPN· *A. aquaticus*^−1^)	<1	<1	<1	6 ± 4	3 ± 2	5 ± 2	1 ± 1	2 ± 1.5

E = *E. coli*, T = total coliform bacteria, total number of analysed *A. aquaticus* = 51, >indicates that the samples contain at least one measurement above the maximum measurable value.

The low degree of association between coliform bacteria and crustaceans was reported also from drinking water distribution systems. Levy *et al.* [5] observed that invertebrates collected from water distribution systems in Worcester, MA (USA) all harboured a variety of bacteria, however, no coliforms were associated with the invertebrates. Likewise, *A. aquaticus* have not been found to carry *E. coli* or other coliform bacteria in drinking water systems [1] and a sampling campaign of zooplankton and amoebae in drinking water did not reveal any associated *E. coli* or other coliform bacteria [25]. Other invertebrates show a higher degree of association with specific bacteria, e.g., copepod associated *Vibrio* sp. reached 7 × 10^4^·copepod^−1^ [26] in spite of copepods being more than ten times smaller than asellids. More than 10^4^ naturally occurring drinking water bacteria (HPC_R2A agar 20 °C_) per *A. aquaticus* have been measured in drinking water samples [21] and *A. aquaticus* host symbiotic bacteria in the hepatopancreases which is not the case for all crustaceans [13]. Hence *A. aquaticus* are able to host bacteria but were not important carriers of *E. coli* or other coliform bacteria though *A. aquaticus* were present in surface water environments with high concentrations of faecal contaminants.

### 3.2. A. aquaticus Associated Coliform Bacteria after Incubation

After incubation for nine weeks at 7 °C, the concentrations of total coliforms increased, while concentrations of *E. coli* decreased (Table 2). Accordingly, no *E. coli* were measured on the animals after nine weeks, while the number of total coliforms per *A. aquaticus* increased substantially, from five MPN per *A. aquaticus* from the pond to 350 MPN per *A. aquaticus* from the beakers. Numbers of *A. aquaticus* associated heterotrophic bacteria in the beakers were 1.3 × 10^5^ CFU·*A. aquaticus*^−1^ (R2A, 20 °C) (Table 2), which is 400 times higher than the numbers of associated coliforms. 

**Table 2:** Concentrations of bacteria from pond 2 in the beaker with the highest bacterial concentrations (September 2010, see Table 1) after nine weeks incubation. Total concentration in the beaker includes sediment and water concentrations.

	*E. coli*	Total coliform bacteria	HPC (R2A, 20 °C)
Total conc. in beaker (MPN·mL^−1^)	0.4	>170	NA
Water phase (MPN·mL^−1^)	<0.01	58	NA
Sediment phase (MPN·mL^−1^)	4	>1,200	NA
Associated (MPN or CFU· *A. aquaticus*^−1^)	<1	350	1.3 ± 0.09 × 10^5^
Start sediment (MPN or CFU·mL^−1^)	<1	<1	3.3 ± 0.14 × 10^4^

NA = not analysed, HPC = heterotrophic plate counts, total number of analysed *A. aquaticus* = 3, >indicates that the samples contain at least one measurement above the maximum measurable value. Start sediment is drinking water sediment before addition to the beakers.

Concentrations of heterotrophic bacteria in a non-chlorinated drinking water batch experiment, in which drinking water *A. aquaticus* was added, reached 1.8 × 10^4 ^CFU·*A. aquaticus*^−1^ (R2A, 20 °C). This was 700 times higher than the number of associated *Klebsiella pneumoniae* (MPN *A. aquaticus*^−1^), which was the coliform bacterium investigated in the study [21]. The only investigated *A. aquaticus* associated pathogen, *Campylobacter jejuni*, showed an even lower degree of association with *A. aquaticus* [21]. When expressed per mL *A. aquaticus*, HPC was also low (5.9 × 10^5 ^CFU·mL^−1^*A. aquaticus*) compared to microscopic crustaceans, on which bacterial numbers ranged between 9.7 × 10^7^ and 5.6 × 10^8^ CFU·mL^−1 ^crustacean [27]. This indicates that other invertebrates may be more important carriers of bacteria than *A. aquaticus*. 

### 3.3. Intrusion Scenarios

The loss of unaccounted for piped drinking water across the globe often amounts to more than 50% [28]. This number covers a large variability with e.g., the Netherlands and Denmark losing only 6–7% [29,30]. The loss occurs by spontaneous leakage in pipes, couplings, tanks, or during repair works as well as due to accidents during excavations. During these incidents the drinking water may be contaminated when the pressure in the pipes is zero or negative [31]. As a risk, normally unaccounted for, *A. aquaticus* is capable of moving against the flow and may enter the system even during positive pressure. 

Two scenarios of intruding *A. aquaticus* are outlined. In the first, *A. aquaticus* enter a drinking water distribution system together with incoming contaminated water, which would occur during pressure loss in pipes or through cracks in clean water tanks during floodings. In the second scenario, single specimens of *A. aquaticus* enter the system e.g., by crawling against the flow in the water coming from a pipe leakage. The maximum concentrations of *E. coli* and total coliforms from the ponds (Table 1) and after nine weeks of incubation (Table 2) were used in the scenarios. According to the European Council Directive [15], *E. coli* and other coliform bacteria must be undetectable in 100 mL of drinking water. Both *E. coli* and total coliforms were regarded as water quality indicators in the following scenarios.

#### 3.3.1. Scenario 1: Do *A. aquaticus* Contribute to the Transport of *E. coli* and Other Coliforms into Distribution Systems during Contaminations with Incoming Water and *A. aquaticus* Together?

In situations where *A. aquaticus* are transported into distribution systems together with contaminated water, and possibly also suspended sediment, e.g., during bursts or maintenance work, the water and sediment itself may transport coliform bacteria into the system. We assessed the contribution of *E. coli* and total coliforms from *A. aquaticus* compared to the contribution from incoming water and sediment. Total numbers of *E. coli* and total coliforms associated with one *A. aquaticus* equaled the numbers of bacteria in 1 mL incoming water (for *E. coli*) or 6 mL incoming water (for total coliforms) or 0.01 mL (for *E. coli*) or 0.3 mL (for total coliforms) in incoming sediment. Hence, the contaminated water and sediment itself can be considered a greater source of coliforms than the *A. aquaticus* transported with the water and sediment. 

#### 3.3.2. Scenario 2: Does Intrusion of Single *A. aquaticus* Specimens without Inflow of Water Cause Exceedings of Drinking Water Guideline Values for *E. coli* and Total Coliforms?

**Figure 1:**
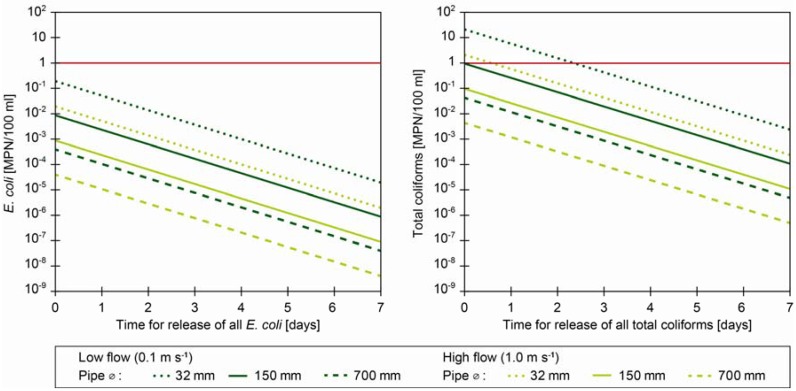
Illustration of *E. coli* and total coliform concentrations in the drinking water if three *A. aquaticus* enter a distribution system carrying the maximum observed concentrations (nine *E. coli* and 1,050 total coliforms). Horizontal lines show European Council Directive guideline values of *E. coli* and total coliforms in drinking water. The effects of the variables: pipe diameter, flow velocity and time for bacterial release, can be read in the illustration.

Normally, it is not considered as an option that bacteria can intrude against the water flow [31] but *A. aquaticus* is able to crawl into a pipe or tank against the flow when water is leaking out through deficiencies in pipes or vents [2], or even crawl short distances without water [10]. Hence, intrusion of *A. aquaticus* is possible without inflow of water. In Scenario 2, three intruding *A. aquaticus* are chosen for the calculations since more animals entering at the same time would most likely be due to a regular inflow of water containing animals, which is described in Scenario 1. In Scenario 2 we have used the maximum observed concentrations of associated bacteria in the ponds and after incubation (three *E. coli* and 350 total coliforms·*A. aquaticus*^−1^) (Table 1, Table 2). In a scenario with discharge of nine *E. coli* and 1,050 total coliform bacteria at once with a perfect mixing of the water, only one litre would contain water with *E. coli* concentrations >1/100 mL, while 110 L would contain water with total coliform concentrations >1/100 mL. This rough estimate provides an overview of the magnitude of the contamination, but is highly simplified and disregards that perfect mixing of the water does not take place in the pipes as well as a simultaneous discharge of all bacteria only occur in rare instances. To elaborate on the simple version of Scenario 2, the following parameters are considered:

(1) All associated bacteria are not likely to be released from *A. aquaticus* instantly. Based on previous experiments we assume that they will be released over time, most likely days [12,21]. We have applied a variable, describing a uniform release over a time frame of one minute to seven days for total release of all coliform bacteria (Figure 1). However, due to dispersion, sedimentation and inactivation, not accounted for in this scenario, the approach is conservative and coliform concentrations are expected to be reduced further.
(2) Pipe diameters of transmission and distribution pipes in most distribution systems vary by an order of magnitude (40–700 mm with an average of 150 mm in a large Danish distribution system). Pipes of 32–40 mm are typically connection pipes to private premises.
(3) The flow rates in distribution systems vary according to the water demands and may even be stagnant e.g., during night. We have calculated the release of bacteria at two flow velocities (0.1 and 1 m·s^−1^).

The three variables influencing *E. coli* and total coliform concentrations in the water were defined as: (1) pipe diameter, (2) flow velocity of the water and (3) time for release of all bacteria.

Drinking water guideline values for *E. coli* were not exceeded in any of the depicted scenarios (Figure 1). Nor did total coliform concentrations exceed guideline values in any scenarios with pipe diameters above 150 mm, but could surpass them in pipes with a diameter below 150 mm, when flow rates are low and all coliforms are released within a short period of time. This situation resemble incidents in which *A. aquaticus* intrude connection pipes where also the distance to the consumer is short and thereby also the time for decay of the bacteria. Unfortunately, many bursts occur in connection pipes, which call for special attention on working procedures concerning pipe work on connection pipes, where flushing through taps after repairs does not provide sufficiently high flow rates to flush out *A. aquaticus* [1]. However, even in these instances it is unlikely to detect *A. aquaticus* associated faecal indicator bacteria during routine monitoring of microbial drinking water quality due to the low sampling frequency.

## 4. Conclusions

*A. aquaticus* from surface water were demonstrated to carry high numbers of heterotrophic bacteria while the share of *E. coli* and other coliform bacteria was low. Conservative calculations showed that during contamination incidents, transport of *E. coli* and other coliform bacteria by *A. aquaticus* into drinking water distribution systems is not significant compared to transport by water and suspended sediment. Hence, intrusion of *A. aquaticus* is not likely to explain incidences of *E. coli* and total coliform exceeding guideline values in drinking water systems because the numbers of *A. aquaticus* associated coliform bacteria are insignificant. However, it still remains to be investigated whether *A. aquaticus* transport pathogens into drinking water systems in higher or lower numbers than the indicator organisms. The study expands the knowledge base for evaluating incidents with presence of coliform indicators in drinking water.
